# Association between deployment and Gulf War Illness and adverse COVID outcomes in a nationwide cohort of 1990–1991 Gulf Era war veterans in the VA’s Million Veteran Program

**DOI:** 10.1371/journal.pone.0348594

**Published:** 2026-06-15

**Authors:** Donna L. White, Alice B. S. NonoDjotsa, Sarah T. Ahmed, Stephen H. Boyle, J. Michael Gaziano, Andrew D. Thompson, Melissa G. Johnson, Elizabeth J. Gifford, Renato Polimanti, Mihaela Aslan, Elizabeth R. Hauser, Drew A. Helmer, on behalf of the Million Veterans Program (MVP)

**Affiliations:** 1 VA HSR&D Center for Innovations in Quality, Effectiveness and Safety (IQuESt), Michael E. DeBakey VA Medical Center, Houston, Texas, United States of America; 2 Department of Medicine, Baylor College of Medicine, Houston, Texas, United States of America; 3 Graduate School of Biomedical Sciences, Baylor College of Medicine, Houston, Texas, United States of America; 4 Center for Translational Research in Inflammatory Diseases (CTRID), Michael E. DeBakey VA Medical Center, Houston, Texas, United States of America; 5 VA Cooperative Studies Program Epidemiology Center—Durham, Department of Veterans Affairs, Durham, North Carolina, United States of America; 6 Massachusetts Veterans Epidemiology Research and Information Center (MAVERIC), VA Boston Healthcare System, Boston, Massachusetts, United States of America; 7 Department of Medicine, Brigham and Women’s Hospital, Harvard Medical School, Boston, Massachusetts, United States of America; 8 Center for Child and Family Policy, Duke Margolis Center for Health Policy, Duke University Sanford School of Public Policy, Durham, North Carolina, United States of America; 9 Cooperative Studies Program Clinical Epidemiology Research Center (CSP-CERC), VA Connecticut Healthcare System 151B, West Haven, Connecticut, United States of America; 10 Department of Psychiatry, Yale School of Medicine, Yale University, New Haven, Connecticut, United States of America; 11 Department of Medicine, Yale School of Medicine, Yale University, New Haven, Connecticut, United States of America; 12 Computational Biology and Bioinformatics Program, Duke University, Durham, North Carolina, United States of America; Mayo Clinic College of Medicine and Science, UNITED STATES OF AMERICA

## Abstract

**Background:**

U.S. military veterans deployed to the Persian Gulf to serve in the 1990–1991 Gulf War (GWVs) have expressed concerns about potential increased risk of experiencing adverse COVID-related health outcomes given broad near-unique exposure to multiple diverse toxic agents during deployment and ongoing ~30% prevalence of Gulf War Illness (GWI+), a chronic, medically unexplained multi-symptom disorder associated with inflammation and immune dysregulation.

**Methods:**

We conducted a retrospective study in the largest nationwide research cohort of 1990−1991Gulf War era veterans (GWEVs) enrolled in the VA’s nationwide Million Veteran Program (MVP) to interrogate: 1) if deployed GWVs had increased risk for testing positive for novel SARS-CoV-2 infection (COVID+), COVID-related hospitalization, ICU admission, or death in 2020 (pre-vaccine period); and 2) if deployed GWVs with GWI^+^ had particularly increased susceptibility. COVID outcomes ascertained using VA’s COVID Data Resource; GWI^+^ determined using CDC severe criteria in surveyed GWV subcohort.

**Findings:**

Our overall GWEV cohort (N = 136,868) had mean age 60.7 years, with 84% male, 27% African-American, and 22% deployed; n = 26,141 COVID-tested in 2020. COVID test-positivity (COVID^+^) was slightly higher in deployed GWVs (18.8% vs. 17.2% in non-deployed GWEVs, p = 0.005). In multivariable logistic models, there were neither strong nor significant associations between deployment and testing COVID^+^, COVID-related hospitalization or ICU admission (adjusted [adj] ORs ranged from 0.90–1.06, all p-values >0.05), nor significant differences in COVID-related mortality. Preliminary analysis in our pre-pandemic surveyed deployed GWV subcohort (N = 1,643 COVID-tested, 39% GWI^+^, 18.4% testing-COVID^+^, 10.6% COVID^+^ hospitalized) suggested GWI^+^ potentially associated with significantly increased COVID-hospitalization risk (adjOR=2.21, 95%CI: 1.02–4.81, p = 0.04); though no significant excess was observed for COVID-related ICU admission or mortality.

**Conclusions:**

Our findings demonstrated that overall deployment to serve in the 1990–1991 Gulf War was not associated with increased COVID-related health risks among Gulf War era veterans using VA healthcare system in early pre-vaccine pandemic era. Preliminary findings suggesting association between Gulf War Illness and potential increased COVID-related hospitalization risk among deployed Gulf War veterans in the early pandemic era warrants replication and ongoing evaluation to assess if it persists in the era of COVID vaccination, oral anti-COVID medications and new viral variants.

## Background

As of the end of December 2024, the World Health Organization reported >777 million COVID-19 infections and >7.1 million deaths recorded globally since the first COVID-19 cases were reported in mid-December 2019 [1]. The U.S. still leads the world in total confirmed COVID cases (103 million) and deaths (1.2 million) and, although the number of the most serious COVID-related outcomes (hospitalizations, ICU admissions, and deaths) have declined, COVID-19 remained in the top 10 leading causes of death in the U.S. in 2023 [2].

COVID-19 is a globally recognized disease of disparities, disproportionately impacting older adults, race/ethnic minority and socioeconomically marginalized populations, and with comorbidities such as obesity, immune compromise, neurological and respiratory conditions. Inequities were especially apparent among workers in occupations with elevated SARS-CoV-2 exposure risk during the pre-vaccine period. It is not well-established, however, whether occupational cohorts with known substantial exposure to other health hazardous agents faced further increased health risks with SARS-CoV-2 infection.

One historically highly occupationally exposed cohort includes the almost 700,000 U.S. veterans—about one-quarter of all U.S. military personnel at the time—who deployed to the Persian Gulf (PG) to serve in the 1990–1991 Gulf War (GW) as part of a multinational military response to Iraq’s invasion of Kuwait. These deployed Gulf War veterans (GWVs) are well-recognized for experiencing broad and, in some cases, near unique combination of exposure to potentially multiple health hazards during deployment, including extensive inhalation of smoke from more than 650 oil well fires and harmful-sized and environmentally cross-contaminated blowing desert sand, low-level nerve agent (sarin/cyclosarin) exposure during chemical weapon plant demolition, and wide self-administered use of anti-nerve agent pyridostigmine bromide pills as pre-exposure prophylaxis to prevent harm when concerned about possible chemical weapon exposure [3–7]. Others included experimental vaccines (e.g., anthrax), depleted uranium munitions, high dosage of widely applied military-grade pesticides, as well as hazards also common in later military conflicts including post-9/11 deployments to Afghanistan and Iraq, such as open burn pits, occupational chemicals, psychological and physical stress, heat, traumatic brain injury, multiple vaccinations, and local zoonoses.

Soon after returning from PG deployment, the deployed GWV cohort drew national attention and Congressionally statute mandated expert research advisory panels and on-going follow-up to evaluate potential long-term health effects associated with these deployment-related exposures given early, elevated prevalence of several unusual health conditions. In addition to suggestive small clusters of early onset cases of two rapidly fatal neurological diseases, amyotrophic lateral sclerosis (ALS) and brain cancer that are extremely rare and occur almost exclusively in older adults in the general population, this also included large numbers of young recently returned and previously healthy deployed GWVs reporting onset of persistent, debilitating, and medically-unexplained clinical symptoms. They frequently spanned multiple clinical domains, most prominently neurological (e.g., cognitive impairment, pain, fatigue, sleep, autonomic, mood disturbance) and dermatologic, gastrointestinal, respiratory, with symptoms typically beginning during or shortly after deployment [4,8,9]. Now termed Gulf War Illness (GWI^+^), it is estimated that over 30% of deployed GWVs remain affected even decades after the conflict ended with this high burden disorder [ 3,4,10–13], with emerging experimental and pre-clinical evidence supporting a neuro-immune etiology arising from persistent immune dysregulation following neurotoxic deployment exposures [14–16].

We conducted a retrospective study leveraging the largest nationwide research cohort comprised of >136,000 veterans with military service during the 1990−1991 Gulf War era (GWEVs) enrolled in the U.S. Department of Veterans Affairs (VA) Million Veteran Program (MVP) [17] to assess whether deployment to the Gulf War conflict theater or having GWI^+^ were associated with increased risk of developing SARS-CoV-2 infection or having more severe COVID-19 outcomes (hospitalization, ICU admission, death). We purposively restricted our evaluation to the early pandemic-period of 2020, prior to advent and wide-scale availability of COVID vaccines, effective oral anti-COVID pharmacotherapy, widespread immunity, and viral variant emergence to evaluate potential impact of deployment and GWI^+^ on contemporary GWEV cohort’s baseline susceptibility to this novel respiratory pathogen.

## Methods

### Study design and GWV Cohort Identification

Our retrospective parent Gulf War era veteran (GWEV) cohort was originally identified for the VA’s Cooperative Studies Program #2006 (CSP2006) Genomics of Gulf War Illness (GWI) study (also known as MVP study #029 [MVP029]) and is well-described elsewhere [18–22]. Briefly, it included >109,000 Department of Defense-confirmed 1990–1991 GWEVs using the VA healthcare system, the largest single integrated nationwide healthcare provider in the U.S. serving >9 million veterans (around half of the ~ 18 million living U.S. veterans). They were identified from among N = 589,620 study eligible veterans enrolled in the VA’s mega-biobank Million Veteran Program (MVP) study between 2011 inception and May 2018. As well-described elsewhere [17], they as MVP participants had written informed consent congruent with the Common Rule obtained at time of MVP recruitment that covers completion of baseline and lifestyle health surveys, provision of blood sample for DNA extraction and genetic testing, linkage with VA’s comprehensive electronic health record (EHR) and administrative healthcare databases for use in MVP approved research, and willingness to be recontacted for future research studies.

We for purposes of the current COVID study subsequently expanded the original parent CSP2006/MVP029 Genomics of GWI study cohort to include >29,000 DoD-confirmed GWEVs newly MVP-enrolled between June 2018 through December 2019. Our final COVID study-eligible expanded cohort was restricted to 136,868 GWEVs confirmed alive as of study start-date 3/1/2020.

Our CSP2006/MVP029 study protocol including amendments to original parent cohort protocol needed to perform current COVID-related research using extant databases with anonymized data was approved by VA’s Central Institutional Review Board (cIRB) subcommittee that oversees all MVP-related research (VA cIRB #19–26). Access to MVP data is limited to eligible VA system users only pursuant to U.S. Department of Veterans Affairs policy. Information regarding VA user eligibility and MVP data application requirements are available at https://vaww.research.va.gov/funding/docs/MVP-Feasibility-Request-Process-for-VA-Investigators.pdf or by contacting MVPLOI@va.gov.

### Identification of GWI in Deployed GWVs in CSP2006 Parent Cohort

As previously described, all eligible original CSP2006 parent cohort members were mailed a one-time 1990–1991 GWEV specific health symptom, medical and military risk factor survey used to identify presence of GWI^+^ between summer 2018-spring 2019 [18,20]. Given absence of a definitive biomarker or diagnostic test, GWI^+^, like other prominent chronic medically unexplained multi-symptom disorders including post-viral chronic fatigue syndrome/myalgic encephalitis (CFS/ME) and long COVID, is clinically diagnosed based on presence of self-reported patient symptom profiles meeting guideline criteria after exclusion of other potential causes.

We used algorithmic methods we previously developed [23] to apply both National Academy of Sciences (NAS)/Institute of Medicine (IOM) recommended gold-standard CDC [8] and Kansas [9] symptom-based research case definitions to 2018 Gulf War survey responses to identify and characterize severity of GWI^+^ in our parent CSP2006 cohort [18–22]. For our current COVID analyses, we *a priori* elected to use only the CDC (Fukuda) chronic multisymptomatic unexplained illness (GWI^+^) criteria to determine GWI^+^ case-status in deployed GWVs to reduce multiplicity of statistical comparisons. We also further limited GWI^+^ to deployed GWVs who met CDC severe criteria to reduce likelihood a potential undiagnosed major medical condition would explain necessary sufficient symptom breadth, burden and duration required to meet diagnostic criteria, while also not excluding those with non-serious and clinically manageable medical conditions that become increasingly prevalent with age (e.g., well-controlled diabetes).

The additional 29,000 GWEVs added to the expanded original parent cohort did not receive the post-enrollment GW survey used to define presence of GWI^+^.

### SARS-CoV2 testing and COVID-19 related health outcomes in 2020

#### Description CSDR Primary COVID Data Source.

We used VA’s COVID Shared Data Resource (CSDR) which since 3/1/2020 has applied large data informatic methods to comprehensive VA electronic healthcare record (EHR) and linked administrative databases to comparably identify and capture specific algorithmically-defined baseline phenotype and COVID-related health variables on all veteran VA healthcare users identified as potentially COVID-infected (i.e., ever-tested for/diagnosed with COVID) [24,25].

#### COVID testing and outcome variables.

We utilized pre-defined CSDR variables to characterize our GWEV cohort members as: 1) Ever-tested if had ≥ 1 SARS-CoV PCR/antigen laboratory test resulting in a confirmed positive/negative result performed in 2020 (and never-tested if no COVID data in CSDR); 2) COVID^±^ if lab test result(s) were ever confirmed COVID^+^, or as COVID^-^ if lab test result(s) only negative; 3) COVID-related hospitalization^±^ if newly hospitalized within ≤14 days of being lab-confirmed COVID^+^; 4) COVID-related ICU admission^±^ if new ICU admission during COVID-hospitalization; and 5) COVID-related death if died ≤30 days of being lab-confirmed COVID^+^.

#### Sociodemographic, Military Service and Clinical Confounding Variables.

We ascertained key sociodemographic (age, race/ethnicity, sex, marital status) and Department of Defense (DoD) confirmed 1990–1991 Gulf War (GW) era military service (PG deployment status, rank, branch, unit component) characteristics for our ~ 29,000 newly added COVID-study eligible GWEVs employing the same VA-DoD merged personnel registry database (VADIR) previously used to comparably characterize them across survey responders and non-responders in our parent cohort [20]. Our final COVID-study eligible expanded CSP2006 cohort included: n = 24,178 deployed GWVs (DoD-confirmed ≥1 day of active military service during 1990–1991 GW era period [August 2, 1990-July 31, 1991] was in the Persian Gulf (PG) conflict theater zone including Iraq, Kuwait, Saudi Arabia, adjacent PG waters/airspace); and n = 86,549 non-deployed GWEVs (DoD-confirmed all 1990–1991 GW era military service occurred only elsewhere). The n = 9,231 DoD-confirmed deployed GWVs parent cohort survey respondents with CDC-classifiable GWI^+^ case-status comprise what is hereafter referred to as our deployed GWV surveyed subcohort used in all GWI^+^ analyses. For all COVID-tested GWEVs (i.e., thus are included in the CSDR), we obtained the following additional *a priori-*specified potential confounders associated with COVID outcomes including in veterans using VA healthcare [26–29] algorithmically captured by the CSDR for the two-year period *prior* to COVID test index date including: Elixhauser comorbidity index [30]; body mass index (BMI); VA healthcare utilization in prior year; non-VA medical co-insurance; geographic region, and lifetime smoking status based on routine VA-mandated health factor screenings.

### Analysis plan

We calculated standard descriptive measures (e.g., mean/%) to summarize our cohort’s sociodemographic and 1990–1991 GW era military service characteristics, both overall and among those ever lab-tested for COVID in 2020; and chi-square/Fisher’s exact and t-tests to interrogate potential differences between deployed GWVs and non-deployed GWEVs and deployed GWI^+^ vs. deployed GWI^-^ GWVs, and respectively.

Regression analyses for CSDR-determined COVID-related testing and outcomes – We utilized well-established epidemiological research practice [31] and methods [32,33] employing a staged hierarchical logistic regression modeling approach with purposive selection of evidence-based *a priori* specified confounders to comparably interrogate whether deployment to 1990–1991 PG conflict theater (in our overall ever COVID-tested cohort), or presence of GWI^+^ (in our ever COVID-tested deployed surveyed subcohort) was associated with increased relative risk for each COVID outcome (i.e., testing COVID^+^, COVID-related hospitalization/ICU admission/death) after accounting for other confounding risk factors.

Our basic approach included performance of univariable analysis examining association between each primary military exposure of interest (e.g., PG deployment in overall GWEV cohort) and all *a priori* specified potential confounders with each individual COVID-related outcome (e.g., testing COVID^+^). This was followed by creation of three tiered multivariable models model including: 1) a *minimal model* adjusting for age, race/ethnicity, gender and Elixhauser comorbidity score, all *a priori* designated as core confounders retained in all multivariable models regardless of statistical significance given biological plausibility and strength of epidemiological evidence [26–29]; 2) a *full (saturated) model* including minimal model covariables plus any of our other *a priori* specified potential confounders (comparably dichotomized to improve model reliability, precision, and stability given sample size related power constraints in deployed GWV subcohort) that had univariable p-values<0.25, a recommended threshold [34] chosen to allow for potential heterogeneity; and 3) a *final reduced (parsimonious) model* retaining minimal model core confounders plus any other potential confounders included in the full model that were retained in final reduced model which was obtained using backward elimination if significant at p < 0.05 level chosen given power constraints and to avoid potential model overfit; with full and iteratively reduced models compared with likelihood ratio tests and Wald tests used for individual covariable significance [32,33]. We used listwise deletion to handle minimal missing data except for geographic region where ~10% missing retained as distinct category. All model results presented as odds ratios (ORs) and associated 95% confidence intervals (CIs).

National Death Index (NDI)-determined COVID mortality in CSP2006 cohort- To determine total COVID-related mortality burden in our entire COVID-study eligible CSP2006 cohort, including the N = 110,727 never-tested and thus without CSDR COVID-related mortality data, we conducted an additional NDI database search to identify all cohort deaths between 3/1/2020-12/31/2020. We classified deaths as COVID-related if ICD-10 diagnostic code for COVID-19 (U07.1) was recorded as the underlying or a contributing cause vs. as death by ‘other cause’. We calculated age-adjusted COVID-related mortality rates/1,000 using the direct method with the overall CSP2006 cohort (N = 136,868) employed as the standard population, and calculated mortality rate ratios (MRRs) and associated 95% CIs to interrogate if adjusted rates significantly differed by deployment status in our overall cohort and by GWI case-status in our deployed surveyed subcohort.

We also performed a novel secondary NDI analysis to assess concordance between VA CSDR identified COVID-related deaths (i.e., death occurred in lab-confirmed COVID^+^ case within 30 days of diagnosis based on then available administrative and electronic health record (EHR)-determined vital status data extracts) vs. was recorded on their official U.S. death certificates available two years later in the NDI datafile.

### Analytic software

All statistical analyses were performed using Python 3.11.7 (https://python.org/).

## Results

### COVID Testing and health outcomes: *Comparison of Deployed vs. Non-Deployed GWVs*

**Fig 1:**
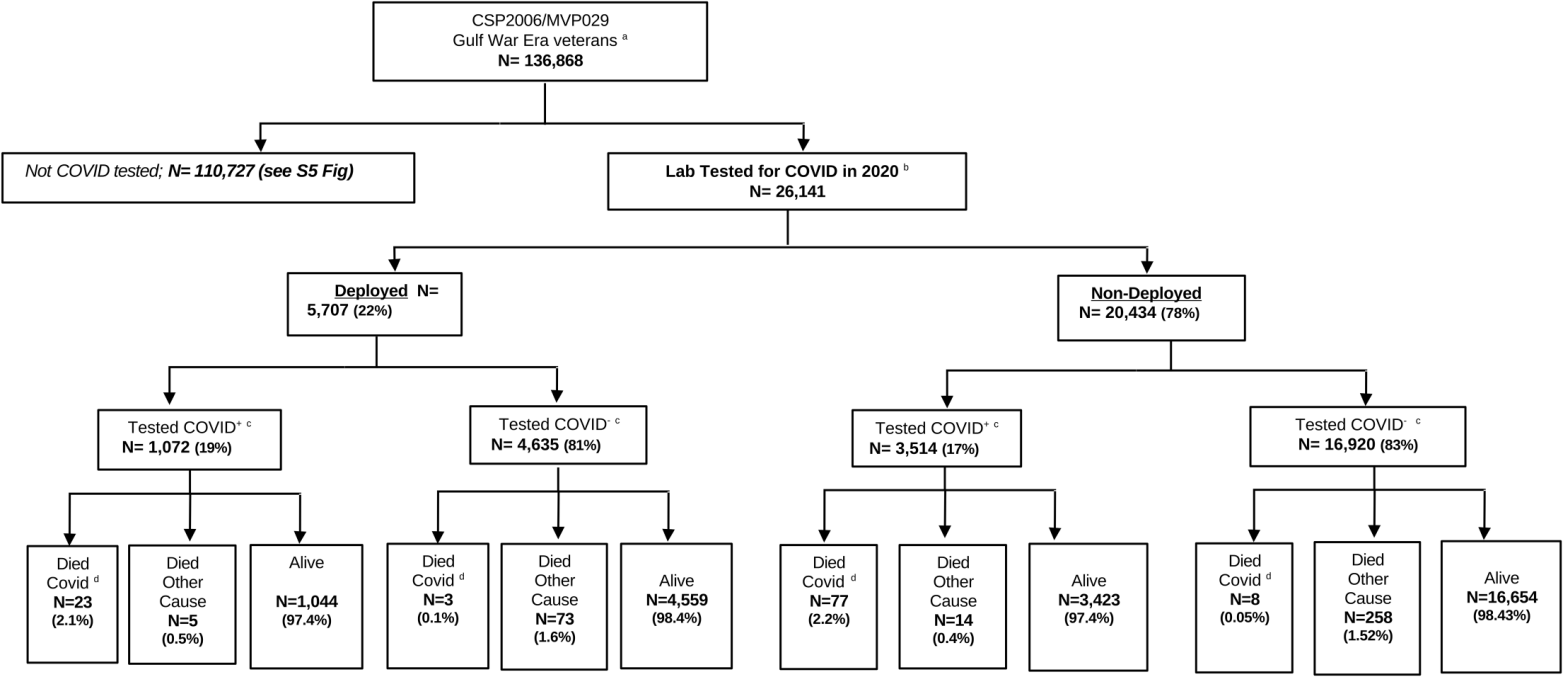
COVID-19 lab-test status and vital outcomes among CSP2006 Gulf War era veterans (GWEVs) ever tested for COVID in 2020, by deployment status. **(A)** Among the *N* = 26,141 GWEVs with a VA-confirmed PCR/antigen COVID test between 3/1/2020 and 12/31/2020, the figure shows branching by deployment status (Deployed *N* = 5,707, 22%; Non-Deployed *N* = 20,434, 78%), COVID test result (COVID^+^ or COVID^−^), and vital outcome (Alive, Died COVID,^d^ or Died Other Cause). The *N* = 110,727 GWEVs not lab-tested for COVID are cross-referenced to S5 Fig. **Superscript footnote definitions:** ^a^ CSP2006 COVID-study eligible expanded cohort of 136,868 DoD-confirmed GWEVs enrolled in MVP by 2020 and confirmed alive as of 3/1/2020. ^b^ VA COVID Shared Data Resource (CSDR) identified as VA-confirmed PCR/antigen laboratory test between 3/1/2020 and 12/31/2020. ^c^ PCR/antigen laboratory test confirmed COVID^+^ if ever tested COVID^+^, and COVID^−^ if all test results confirmed COVID^−^ during the study period. ^d^ National Death Index (NDI) death-certificate match: COVID listed as underlying or contributing cause of death; otherwise coded other cause of death; no match indicates alive through 12/31/2020.

COVID-tested cohort description: Our VA COVID Shared Data Resource (CSDR) searches identified 26,141 (>19%) of our COVID-study eligible CSP2006 Gulf War era veteran (GWEV) cohort members had ≥ 1 VA lab-performed PCR or antigen test with confirmed SARS-CoV-2 test findings between 3/1/2020 and 12/31/2020. (Fig 1) Almost 22% (n = 5,707) were deployed GWVs; deployed a few years younger (mean age: 58.0 vs. 60.6 in non-deployed GWEVs), and modestly more likely male (86.9% vs. 79.7%) and African-American (34.2% vs. 29.7%) than non-deployed GWEVs, all p-values<0.001. (S2 Table) Sociodemographic and military characteristics in our never-tested GWEVs (n = 110,727) were broadly similar with those in ever-tested, with again only modest differences in magnitude (e.g., mean age: 58.6 vs. 61.5 years, African-American: 30.1% vs. 24.6% in never-tested deployed vs. non-deployed GWEVs, respectively, p-values<0.001) (S2 Table).

*COVID-related testing and health outcomes*: *1) Test positivity:* Overall crude COVID test positivity was higher in deployed GWVs (18.8%) vs. 17.2% in non-deployed GWEVs (p < 0.005). (Table 1) Deployed COVID^+^ (n = 1,072) were ~2.2 years younger (mean age: 57.4 vs. 59.6 non-deployed GWEVs), and modestly but significantly more likely to be male (88.4% vs. 83.6%) and African-American (38.2% vs. 31.6%) vs. non-deployed COVID^+^ GWEVs (n = 3,514). (S2 Table) Deployment was associated with significantly increased COVID test-positivity risk (OR_unadj_: 1.11, 95% CI: 1.03–1.20, p = 0.005) in univariable analysis. (Table 2) However, associations were attenuated and non-significant in adjusted tiered-multivariable models (range ORs_adj_: 1.04–1.06, N.S.).

**Table 1:** COVID-related outcomes across deployment and GWI case-status in COVID-tested Gulf War Era Veterans (GWEVs) in 2020.

	Overall CSP2006 GWEV cohort COVID tested in 2020 ^a^				Deployed GWV subcohort COVID tested in 2020 ^b^			
	All	Deployed	Not Deployed		All	GWI- ^c^	GWI + ^c^	
	N = 26,141	n=5,707	n=20,434		N = 1,643	n=998	n=645	
*VA CSDR-determined COVID outcomes* ^*d*^		(21.8%)	(78.2%)	p-value		(60.7%)	(39.3%)	p-value
Lab confirmed COVID^+ e^, n (%)	4586 (17.5)	1072 (18.8)	3514 (17.2)	0.005	303 (18.4)	192 (19.2)	111 (17.2)	0.30
Newly hospitalized within 14 days from lab confirmed COVID ^+^ , n (%)	679 (14.8)	145 (13.5)	534 (15.2)	0.18	32 (10.6)	14 (7.3)	18 (16.2)	0.02
ICU admission during COVID-related 14-day hospitalization, n (%)	220 (32.4)	37 (25.5)	183 (34.3)	0.047	5 (15.6)	2 (14.3)	3 (16.7)	>0.99
Died within 30 days of lab confirmed COVID ^+^ , n (%)	103 (2.2)	24 (2.2)	79 (2.2)	0.99	8 (2.6)	6 (3.1)	2 (1.8)	0.71

Legend:

^a^Identified by VA CSDR as PCR/antigen laboratory COVID-tested by VA from among N = 136,868 expanded CSP2006 GWEV cohort members enrolled in MVP confirmed alive as of 3/1/2020.

^b^Identified by VA CSDR as PCR/antigen laboratory COVID-tested by VA from among N = 1,643 Deployed GWV subcohort from original CSP2006 parent cohort who completed 2018 GW survey to classify GWI case-status.

^c^Determined for earlier published study [18] using application of CDC severe (Fukuda) criteria [8] to GW survey responses to classify as meeting criteria for (GWI^+^) or not (GWI^-^).

^d^Identified using VA Covid Shared Data Resource pre-defined COVID algorithmic phenotypes for study period 3/1/2020-12/31/2020.

^e^COVID^+^ defined if any PCR/antigen lab test VA CSDR confirmed was confirmed definite positive result during study period (3/1/2020-12/31/2020).

Abbreviations:

CDC, Centers for Disease Control and Prevention

COVID+, VA PCR/antigen laboratory test confirmed definite COVID+ test finding between 3/1/2020-12/31/2020

COVID-, VA PCR/antigen laboratory test findings all confirmed definite COVID-

CSP2006, VA Cooperative Studies Program Gulf War Era Veteran (GWEV) Cohort 2006 (also Million Veteran Program (MVP) cohort 029)

CSDR, VA’s COVID Shared Data Resource

GWI+, Gulf War Illness positive for severe CDC GWI criteria; GWI-, Gulf War Illness absent as not meet CDC GWI criteria

GWEV, Gulf War Era Veteran with DoD confirmed military service of any type or location in 1990-1991 Gulf War period

GWV, Gulf War Era veteran deployed to Persian Gulf to serve in 1990-1991 Gulf War

ICU, Intensive Care Unit

PCR, polymerase chain reaction MVP, VA Million Veteran Program

VA, U.S. Department of Veterans Affairs

**Table 2:** Logistic regression models assessing 1990–1991 Gulf War deployment and Gulf War Illness and COVID-related outcomes in GWEVs in pre-vaccine pandemic period.

Source/ Cohort	Comparison	Model	OR (95% CI)	p-value	Adjustment Covariables^e,f^
**A) Testing COVID+** ^b^					
**Overall CSP2006 GWEV cohort (N = 26,141)** ^a^	*Deployed vs. Non-Deployed*	Univariable	1.11 (1.03–1.20)	0.005	NA
		Minimal	1.04 (0.96–1.13)	0.36	Age, sex, race/ethnicity, Elixhauser comorbidity score
		Full (saturated)ᶜ	1.07 (0.99–1.15)	0.10	Age, sex, race/ethnicity, Elixhauser, marital status, service branch, unit component, smoking, BMI, co-insurance, geographic region
		Final (reduced)ᵈ	1.06 (0.99–1.16)	0.12	Age, sex, race/ethnicity, Elixhauser, marital status, unit component, BMI
**Deployed GWV Subcohort (N = 1,643)** ^j^	*GWI + vs. GWI −* ^k^	Univariable	0.87 (0.67–1.13)	0.30	NA
		Minimal	0.85 (0.65–1.11)	0.23	Age, sex, race/ethnicity, Elixhauser comorbidity score
		Full (saturated)ᶜ	0.84 (0.64–1.11)	0.24	Age, sex, race/ethnicity, Elixhauser, marital status, unit component, BMI, smoking, co-insurance, geographic region
		Final (reduced)ᵈ	0.82 (0.62–1.07)	0.26	Age, sex, race/ethnicity, Elixhauser, marital status, unit component, BMI, smoking
**B) COVID-related hospitalization** ^g^					
**Overall CSP2006 GWEV cohort (N = 26,141)** ^a^	*Deployed vs. Non-Deployed*	Univariable	0.87 (0.72–1.06)	0.18	NA
		Minimal	0.91 (0.73–1.12)	0.37	Age, sex, race/ethnicity, Elixhauser comorbidity score
		Full (saturated)ᶜ	0.90 (0.72–1.12)	0.35	Age, sex, race/ethnicity, Elixhauser, marital status, unit component, rank, BMI, co-insurance, geographic region
		Final (reduced)ᵈ	0.90 (0.74–1.11)	0.34	Age, sex, race/ethnicity, Elixhauser, marital status, co-insurance
**Deployed GWV Subcohort (N = 1,643)** ^j^	*GWI + vs. GWI −* ^k^	Univariable	2.46 (1.17–5.16)	0.02	NA
		Minimal	2.21 (1.01–4.81)	0.04	Age, sex, race/ethnicity, Elixhauser comorbidity score
		Full (saturated)ᶜ	2.13 (0.96–4.72)	0.05	Age, sex, race/ethnicity, Elixhauser, marital status, unit component, geographic region
		Final (reduced)ᵈ	2.21 (1.01–4.81)	0.04	Age, sex, race/ethnicity, Elixhauser
**C) COVID-related ICU admission** ^h^					
**Overall CSP2006 GWEV cohort (N = 26,141)** ^a^	*Deployed vs. Non-Deployed*	Univariable	0.66 (0.43–0.99)	0.047	NA
		Minimal	0.72 (0.47–1.10)	0.13	Age, sex, race/ethnicity, Elixhauser comorbidity score
		Full (saturated)ᶜ	0.74 (0.48–1.14)	0.17	Age, sex, race/ethnicity, Elixhauser, rank, smoking, BMI, co-insurance, branch, unit component, geographic region
		Final (reduced)ᵈ	0.72 (0.47–1.10)	0.13	Age, sex, race/ethnicity, Elixhauser
**Deployed GWV Subcohort (N = 1,643)** ^j^	*GWI + vs. GWI −* ^k^	Univariable & Multivariable	NRˡ	—	Not modeled—insufficient event numbers in deployed GWV subcohort
**D) COVID-related mortality** ^i^					
**Overall CSP2006 GWEV cohort (N = 26,141)** ^a^	*Deployed vs. Non-Deployed*	Univariable	0.99 (0.63–1.58)	0.99	NA
		Minimal	1.27 (0.79–2.05)	0.32	Age, sex, race/ethnicity, Elixhauser comorbidity score
		Full (saturated)ᶜ	1.19 (0.72–1.94)	0.49	Age, sex, race/ethnicity, Elixhauser, rank, smoking, BMI, co-insurance, branch, unit component, geographic region
		Final (reduced)ᵈ	1.27 (0.79–2.05)	0.32	Age, sex, race/ethnicity, Elixhauser
**Deployed GWV Subcohort (N = 1,643)** ^j^	*GWI + vs. GWI −* ^k^	Univariable & Multivariable	NRˡ	—	Not modeled—insufficient event numbers in deployed GWV subcohort

**Legend:**

^a^VA COVID Shared Data Resource (CSDR) identified PCR/antigen laboratory COVID-tested GWEVs in expanded CSP2006/MVP029 cohort (N = 136,868 DoD-confirmed 1990–1991 Gulf War veterans) between 3/1/2020 and 12/31/2020.

^b^VA PCR/antigen lab-confirmed COVID+ between 3/1/2020–12/31/2020, vs. COVID− if always confirmed COVID− in study period.

^c^Includes minimal model core confounders (age, Elixhauser, race/ethnicity, sex) plus any other variables with univariable p < 0.25.

^d^Includes minimal model covariables plus any other confounders retained from full model at p < 0.05 after backward elimination.

^e^Minimal missing data handled with listwise deletion; geographic region (~10% missing) retained as separate unknown category.

^f^Non-binary categorical confounders dichotomized to improve model stability given sample size constraints [e.g., Branch: Army vs. Other; Region: South vs. Non-South].

^g^VA CSDR-defined new hospitalization within 14 days of lab-confirmed COVID + .

^h^ICU admission during COVID-related hospitalization.

^i^Death within 30 days of lab-test confirmed COVID+ (in hospital and community).

^j^Deployed GWV surveyed subcohort members with known GWI case-status who had ≥ 1 VA-confirmed PCR/antigen COVID test result between 3/1/2020–12/31/2020.

^k^GWI case status defined using CDC (Fukuda) severe criteria applied to 2018 GW survey responses.

^l^Not reported—not modeled due to low event numbers in deployed GWV subcohort.

Abbreviations: CDC, Centers for Disease Control and Prevention; CI, Confidence Interval; COVID + , SARS-CoV-2 PCR/antigen lab-confirmed positive; CSP2006, VA Cooperative Studies Program Genomics of Gulf War Illness Study Cohort (also MVP029); DoD, U.S. Department of Defense; GWI, Gulf War Illness; GWEV, DoD-confirmed 1990–1991 Gulf War era veteran; GWV, DoD-confirmed GWEV deployed to PG conflict theater; MVP, Million Veteran Program; NR, not reported; OR, odds ratio; PCR, polymerase chain reaction; PG, Persian Gulf; VA, U.S. Department of Veterans Affairs.

1) *COVID-related hospitalization:* Unadjusted COVID-related hospitalization prevalence was low and non-significantly different by deployment status (13.5% in deployed GWVs vs. 15.2% in non-deployed GWEVs, p = 0.18). (Table 2) Deployment was similarly not associated with COVID-related hospitalization risk across tiered-multivariable models (ORs_adj_ range: 0.87–0.90, all p-values≥0.23). (Table 2)
2) *COVID-related ICU admission:* Crude ICU admission prevalence during COVID-hospitalization was significantly *lower* in deployed GWVs (25.5% vs. 34.3% in non-deployed GWEVs, p = 0.047, Table 1), with significant reduced relative ICU admission risk in univariable analysis (OR_unadj_ = 0.66, 95% CI: 0.43–0.99). (Table 2) However, effects were attenuated and non-significant in all tiered-multivariable models (e.g., OR_adj___final_ = 0.72, 95% CI: 0.47–1.10, p = 0.13).
3) *COVID-related 30-day mortality:* There were 103 COVID-related deaths among 4,586 lab-confirmed COVID^+^ GWEVs, with 2.2% of both deployed GWVs and non-deployed GWEVs dying within 30 days of test-positivity. (Table 1) Deployment was not significantly associated with COVID-related mortality in univariable (OR_unadj_ = 0.99, p > 0.99) through final multivariable analysis (OR_adj_final_ = 1.27, 95% CI: 0.79–2.05, p = 0.32). (Table 2)

### COVID testing and health outcomes: *Comparison of GWI*^*+*^ *vs. GWI*^*-*^ *in deployed surveyed sub-cohort*

COVID-tested deployed sub-cohort description: There were 1,643 deployed GWV sub-cohort members (n = 645 [35%] meeting CDC severe GWI criteria [GWI^+^] and n = 998 [65%] GWI^-^) who were COVID-tested in 2020. (S5 Fig) COVID-tested deployed GWVs who were GWI^+^ were ~1.3 years younger (mean = 59.0 vs. 60.3 GWI^-^), modestly more likely to be non-White (e.g., Black: 33.5% vs. 22.9%) than ever-tested deployed GWVs who were GWI^-^, p-values<0.001. (S2 Table)

COVID-related testing and health outcomes: 1) *Test positivity*: Among 303 deployed sub-cohort members who tested COVID^+^, 37% (n = 111) were GWI^+^, with GWI^+^ ~ 1 year younger than both COVID^+^ GWI^-^ and also GWVs who were GWI^+^ but tested only COVID^-^ (mean age: 57.9 COVID^+^ GWI^+^ vs. 59.0 and 59.2 years in COVID^+^ GWI^-^ and COVID^-^ GWI^+^, respectively). (S2 Table) Overall crude COVID test positivity was non-significantly 2% lower in GWI^+^ (17.2% vs. 19.2% GWI^-^). (Table 1) GWI^+^ was not associated with relative COVID+ risk across tiered-multivariable models (e.g., OR_adj_final_reduced_ = 0.82, 95% CI: 0.62–1.07, p = 0.15). (Table 2)

2) *COVID-related hospitalization*: Approximately 10% (n = 32) of COVID^+^ deployed GWV subcohort members were hospitalized within 14 days, with significantly higher crude prevalence observed in deployed GWI^+^ (16.2% vs 7.3% in deployed GWI^-^, p = 0.02). (Table 1) GWI^+^ was associated with a suggestive >2-fold increased relative COVID-related hospitalization risk across all tiered logistic models (e.g., OR_unadj_ = 2.46, p = 0.02; OR_adj_final_parsimonious_ = 2.20, p = 0.04). (Table 2) We found similar suggestive over two-fold significant increased relative risk in *post hoc* multivariable sensitivity analyses where we further dichotomized non-binary core confounders (Age, Elixhauser, Race/Ethnicity) to further increase model reliability and precision, and in sub-group analysis limited to males only. (data not shown)

### *Post-hoc* exploratory evaluation of clinical comorbidities in association between GWI^±^ and COVID-hospitalization risk

We added a secondary *post-hoc* hypothesis generating descriptive evaluation to explore if any of the 15 most prevalent individual clinical factors included in Elixhauser comorbidity index [ 30] or among CDC evidence-based review conditions associated with severe COVID risk [26] may be selectively associated with our suggestive preliminary finding of >2-fold potential excess relative COVID-related hospitalization risk among deployed GWVs who were GWI^+^. We employed well-established epidemiological practice including p-value threshold of p < 0.20 [ 34] without further correction for multiple comparisons [ 35,36] to guide descriptive exploratory identification of potential suggestive additional confounders to prioritize for examination in future large sample hypothesis testing research.

We found three conditions suggestively potentially more common in deployed GWI^+^ who were COVID-hospitalized vs. similarly COVID^+^ deployed GWVs also COVID-hospitalized but who were GWI^-^; depression (occurred in 13 of 18 [72%] GWI^+^ COVID-hospitalized vs. 3 of 14 [21%] GWI^-^ COVID-hospitalized, p = 0.01); bronchitis (in 5 of 18 [28%] GWI^+^ but in 0 of 14 GWI^-^, p = 0.05); and coronary atherosclerotic heart disease [CAHD] (also in 5 of 18 [28%] GWI^+^ but in 0 of 14 GWI^-^, p = 0.05). (Table 3)

**Table 3:** *Post-hoc* descriptive comparison of comorbid risk factors in COVID+ deployed Gulf War Veterans according to COVID-hospitalization and GWI case-status.

*Comorbidity*^*d*^:		Deployed Subcohort GWVs Confirmed COVID^+^ (N = 303)^a^							
		Hospitalized for COVID ^b^				Not hospitalized for COVID			
		Total N = 32	GWI^- c^ n = 14	GWI ^+^ ^c^ n = 18	P-Value	Total N = 271	GWI^- c^ n = 178	GWI ^+^ ^c^ n = 93	P-Value
Asthma	No	28 (87.5)	12 (85.7)	16 (88.9)	>0.99	246 (90.8)	164 (92.1)	82 (88.2)	0.40
	Yes	4 (12.5)	2 (14.3)	2 (11.1)		25 (9.2)	14 (7.9)	11 (11.8)	
Bronchitis	No	27 (84.4)	14 (100.0)	13 (72.2)	0.05	250 (92.3)	163 (91.6)	87 (93.5)	0.74
	Yes	5 (15.6)	0 (0)	5 (27.8)		21 (7.7)	15 (8.4)	6 (6.5)	
COPD	No	27 (84.4)	13 (92.9)	14 (77.8)	0.36	250 (92.3)	166 (93.3)	84 (90.3)	0.54
	Yes	5 (15.6)	1 (7.1)	4 (22.2)		21 (7.7)	12 (6.7)	9 (9.7)	
Cancer	No	29 (90.6)	13 (92.9)	16 (88.9)	>0.99	234 (86.3)	155 (87.1)	79 (84.9)	0.77
	Yes	3 (9.4)	1 (7.1)	2 (11.1)		37 (13.7)	23 (12.9)	14 (15.1)	
Coronary atherosclerotic heart	No	27 (84.4)	14 (100.0)	13 (72.2)	0.05	241 (88.9)	160 (89.9)	81 (87.1)	0.62
disease	Yes	5 (15.6)	0 (0)	5 (27.8)		30 (11.1)	18 (10.1)	12 (12.9)	
Cerebrovascular disease	No	31 (96.9)	14 (100.0)	17 (94.4)	>0.99	267 (98.5)	176 (98.9)	91 (97.8)	0.61
	Yes	1 (3.1)	0 (0)	1 (5.6)		4 (1.5)	2 (1.1)	2 (2.2)	
Chronic Neuromuscular Disease	No	30 (93.8)	14 (100.0)	16 (88.9)	0.49	268 (98.9)	176 (98.9)	92 (98.9)	>0.99
	Yes	2 (6.2)	0 (0)	2 (11.1)		3 (1.1)	2 (1.1)	1 (1.1)	
Liver Cirrhosis	No	32 (100.0)	14 (100.0)	18 (100.0)	>0.99	268 (98.9)	175 (98.3)	93 (100.0)	0.55
	Yes	0 (0)	0 (0)	0 (0)		3 (1.1)	3 (1.7)	0 (0)	
Chronic Kidney Failure	No	31 (96.9)	14 (100.0)	17 (94.4)	>0.99	267 (98.5)	176 (98.9)	91 (97.8)	0.61
	Yes	1 (3.1)	0 (0)	1 (5.6)		4 (1.5)	2 (1.1)	2 (2.2)	
Dementia	No	31 (96.9)	14 (100.0)	17 (94.4)	>0.99	267 (98.5)	174 (97.8)	93 (100.0)	0.30
	Yes	1 (3.1)	0 (0)	1 (5.6)		4 (1.5)	4 (2.2)	0 (0)	
Type 2 Diabetes	No	15 (46.9)	6 (42.9)	9 (50.0)	0.96	172 (63.5)	117 (65.7)	55 (59.1)	0.35
	Yes	17 (53.1)	8 (57.1)	9 (50.0)		99 (36.5)	61 (34.3)	38 (40.9)	
Hypertension	No	6 (18.8)	3 (21.4)	3 (16.7)	>0.99	86 (31.7)	60 (33.7)	26 (28.0)	0.41
	Yes	26 (81.2)	11 (78.6)	15 (83.3)		185 (68.3)	118 (66.3)	67 (72.0)	
Major depressive disorder	No	16 (50.0)	11 (78.6)	5 (27.8)	0.01	160 (59.0)	129 (72.5)	31 (33.3)	<0.001
	Yes	16 (50.0)	3 (21.4)	13 (72.2)		111 (41.0)	49 (27.5)	62 (66.7)	
Alcohol dependence history	No	27 (84.4)	13 (92.9)	14 (77.8)	0.36	200 (73.8)	128 (71.9)	72 (77.4)	0.40
positive	Yes	5 (15.6)	1 (7.1)	4 (22.2)		71 (26.2)	50 (28.1)	21 (22.6)	
Anxiety disorder	No	25 (78.1)	12 (85.7)	13 (72.2)	0.43	209 (77.1)	143 (80.3)	66 (71.0)	0.11
	Yes	7 (21.9)	2 (14.3)	5 (27.8)		62 (22.9)	35 (19.7)	27 (29.0)	
Drug dependency history	No	31 (96.9)	14 (100.0)	17 (94.4)	>0.99	263 (97.0)	175 (98.3)	88 (94.6)	0.13
positive	Yes	1 (3.1)	0 (0)	1 (5.6)		8 (3.0)	3 (1.7)	5 (5.4)	
Chronic kidney disease	No	27 (84.4)	13 (92.9)	14 (77.8)	0.36	255 (94.1)	168 (94.4)	87 (93.5)	>0.99
	Yes	5 (15.6)	1 (7.1)	4 (22.2)		16 (5.9)	10 (5.6)	6 (6.5)	
Obesity (BMI ≥ 30)	No	11 (34.3)	5 (35.7)	6 (33.4)	0.98	97 (35.6)	68 (38.4)	28 (31.1)	0.51
	Yes	21 (65.6)	9 (64.3)	12 (66.6)		174 (64.4)	109 (61.6)	65 (69.9)	
Smoking history positive	No	11 (37.5)	5 (35.7)	7 (38.9)	0.46	142 (53.8)	93 (54.1)	49 (53.3)	>0.99
	Yes	20 (62.5)	9 (64.3)	11 (61.1)		122 (46.2)	79 (45.9)	43 (46.7)	
Non-White race/ethnicity	No	14 (43.8)	7 (50.0)	7 (38.9)	0.41	162 (59.8)	122 (68.5)	40 (43.0)	<0.001
	Yes	18 (56.2)	7 (50.0)	11 (61.1)		109 (40.2)	56 (31.5)	53 (57.0)	
Male gender	No	3 (9.4)	2 (14.3)	1 (5.6)	0.57	28 (10.3)	17 (9.6)	11 (11.8)	0.71
	Yes	29 (90.6)	12 (85.7)	17 (94.4)		243 (89.7)	161 (90.4)	82 (88.2)	
Age (in years, 3/2020)	Mean (SD)	57.4 (6.6)	58.3 (7.4)	56.4 (5.6)	0.41	58.8 (7.6)	59.2 (7.6)	57.9 (7.4)	0.17
Elixhauser Comorbidity Score	No	20 (52.5)	9 (64.3)	11 (61.1)	>0.99	216 (79.7)	142 (79.7)	74 (79.6)	>0.99
(Upper Tertile >18)	Yes	12 (37.5)	5 (35.7)	7 (38.9)		55 (20.3)	36 (20.2)	19 (20.4)	

Legend:

^a^DoD-confirmed deployed GWV subcohort member who completed 2018 GW survey and VA CSDR PCR/antigen lab test confirmed COVID+ between 3/1/2020 and 12/31/2020.

^b^New COVID-related hospitalization VA CSDR defined as within 14 days of lab-confirmed COVID+ test.

^c^GWI case status determined using CDC (Fukuda) severe symptom-based criteria [8] based on 2018 GW Survey responses; GWI^+^ meets criteria, otherwise GWI^-^ [18].

^d^VA CSDR algorithmic extraction from complete VA EHR databases for 15 most prevalent conditions in Elixhauser comorbidity index [30] or CDC evidence review [26] severe COVID risk

^f^actors using pre-specified phenotype definitions for period spanning two years prior to COVID index test date.

Abbreviations:

CDC, Centers for Disease Control and Prevention

COPD, chronic obstructive pulmonary disease

COVID+, VA PCR/antigen laboratory test confirmed definite COVID+ test finding between 3/1/2020-12/31/2020

COVID-, VA PCR/antigen laboratory test findings all confirmed definite COVID-

CSDR, VA’s COVID Shared Data Resource

DoD, U.S. Department of Defense

EHR, electronic health record

GWI+, Gulf War Illness positive for severe CDC GWI criteria

GWI-, Gulf War Illness absent as not meet CDC GWI criteria

GWV, DoD-confirmed deployed Gulf War Era Veteran (GWEV)

VA, U.S. Department of Veterans Affairs

Depression, however, was similarly highly prevalent among COVID^+^ GWI^+^ who were not ultimately hospitalized for COVID (occurring in 62 of 93 [67%] vs. 72% in GWI^+^ who were COVID-hospitalized, N.S.). In contrast, both bronchitis and CAHD were also suggestively potentially differentially more prevalent in deployed COVID^+^ GWI^+^ who were COVID-hospitalized vs. COVID^+^ GWI^+^ who were not ultimately hospitalized for COVID (bronchitis: 28% in GWI^+^ COVID hospitalized vs. 7% GWI^+^ not hospitalized, p = 0.02; and CAHD: 28% GWI^+^ hospitalized vs. 13% GWI^+^ not hospitalized, p = 0.14, respectively). (Table 3) Although we cannot distinguish type of bronchitis based on CSDR-identified bronchitis (which captures both chronic and acute bronchitis diagnostic codes) given inability to perform confirmatory chart review within the VA’s CSDR framework, 4 of 5 also had COPD diagnostic codes, suggesting multiple bronchitis cases may be chronic.

3) *COVID-related ICU admission:* Only 5 deployed COVID^+^ sub-cohort members hospitalized for COVID were subsequently ICU-admitted, with similar non-significantly different crude ICU prevalence rates (16.6% GWI^+^ vs. 14.3% GWI^-^, respectively). (Table 1) Limited numbers precluded modeling.
4) *COVID-related mortality:* There were only 8 deaths within 30 days of testing lab-confirmed COVID^+^, with non-significantly different low crude COVID-related mortality (1.8% in deployed GWI^+^ vs. 3.1% in GWI^-^, p = 0.71). (Table 2) Limited numbers precluded modeling.

### National Death Index-determined overall and COVID-related mortality burden in GWVs in 2020

Study flow diagram Fig 1 includes final National Death Index (NDI) death-certificate determined vital status in our COVID-tested GWEV cohort as of the end of the study period, 12/31/2020. See Supplemental Results for documenting excellent concordance between NDI-determined COVID mortality and VA CSR&D 30-day COVID mortality, and comparative impact on overall survival in the 10-month early pandemic period in 2020 vs. year prior. (S4 Text)

#### COVID mortality burden associated with Deployment:.

There were N = 207 CSP2006 GWEV cohort members who died with COVID as death certificate-recorded cause of death; including n = 100 (48%) among 4,586 lab-confirmed COVID^+^ GWEVs (Fig 1) and n = 96 (46%) among 110,727 GWEVs never COVID-tested in 2020. (S5 Fig)

Of the *N* = 110,727 GWEVs not lab-tested for COVID (cross-referenced from Fig 1), the figure branches by deployment status (Deployed *N* = 24,178, 22%; Non-Deployed *N* = 86,549, 78%) and vital outcome (Alive, Died COVID,^d^ or Died Other Cause) through 12/31/2020. The ever COVID-tested subcohort (*N* = 26,141) is cross-referenced to Fig 1. Footnote definitions are as in Fig 1.

Overall deployment to serve in 1990–1991 PG conflict theater zone was not associated with any death-certificate based metric of COVID mortality burden: with extremely low proportions of both deployed GWVs and non-deployed GWEVs who never received a VA-lab confirmed COVID^+^ diagnosis ultimately dying with death certificate-recorded COVID as a cause of death in 2020 (e.g., 0.1% in both never-tested deployed GWVs and non-deployed GWEVs, respectively) (Table 4). Considerably higher though also nearly identical proportions of lab-confirmed COVID^+^ deployed GWVs and non-deployed GWEVs died of COVID (2.2% vs. 2.1%, respectively, N.S.), with COVID accounting for most of all deaths observed in COVID^+^ GWEVs during our 10-month study period (82.1% in deployed, 76.2% in non-deployed, Table 4, N.S.). There were also similar overall age-adjusted COVID mortality rates among deployed GWVs and non-deployed GWEVs (e.g., 24.16/1,000 and 23.98/1,000 in lab-confirmed COVID^+^ deployed and non-deployed, respectively; MRR = 1.01, 95% CI: 0.54–1.47, N.S.) (S3 Table).

**Table 4:** National Death Index-determined COVID-related mortality burden in nationwide CSP2006/MVP029 Gulf War Era Veteran Cohort in 2020.

4A. In overall CSP2006 COVID study eligible GWEV cohort by deployment and COVID testing status (N = 136,868)																			
	Ever COVID tested^a^ in 2020 overall GWEV cohort (N = 26,141)													Never COVID tested in 2020 (N = 110,727)					
	COVID^+^ *(n = 4,586)*						COVID^-^ *(n = 21,555)*							Deployed			Not Deployed		
	Deployed			Not Deployed			Deployed			Not Deployed				n = 24,178			n = 86,549		
	(n = 1,072)			(n = 3,514)			(n = 4,635)			(n = 16,920)									
		Age (yrs)			Age (yrs)				Age (yrs)			Age (yrs)			Age (yrs)				Age (yrs)
NDI-determined vital status thru 2/31/2020^b^	n (%)	Mean (SD)		n (%)	Mean (SD)		n (%)		Mean (SD)	n (%)		Mean (SD)		n (%)	Mean (SD)		n (%)		Mean (SD)
Deceased-COVID	23 (2.1)	61.3 (7.9)		77 (2.2)	66.6 (9.2)		3 (0.1)		61.8 (8.4)	8 (0.05)		64.4 (8.6)		17 (0.1)	63.6 (5.7)		79 (0.1)		70.7 (8.2)
Deceased-Other cause	5 (0.5)	66.0 (9.0)		14 (0.4)	68.1 (11.2)		73 (1.6)		58.1 (7.0)	285 (1.52)		64.5 (8.4)		182 (0.7)	62.0 (8.7)		790 (0.9)		67.0 (9.5)
Alive through 12/31/2020	1,044 (97.4)	57.3 (6.9)		3,423 (97.4)	59.5 (7.8)		4,559 (98.3)		62.3 (5.0)	16,654 (98.43)		60.7 (7.9)		24,144 (99.2)	58.5 (7.3)		85,680 (99.0)		61.4 (8.5)
Proportion total deaths due to COVID	82.1%			76.2%			3.9%			2.6%				8.5%			9.1%		
**4B. In Deployed COVID-tested GWV subcohort by GWI case-status**^**c**^ **(N = 9,231)**																			
	Ever COVID tested^a^ Deployed subcohort in 2020 (N = 1,643)																		
	COVID^+^ (n = 303)										COVID^-^ (n = 1,340)								
	GWI^+^					GWI^-^					GWI^+^					GWI^-^			
	n = 111					n = 192					n = 534					n = 806			
			Age (yrs)					Age (yrs)					Age (yrs)					Age (yrs)	
NDI-determined vital status 3/1/2020-12/31/2020^b^	n (%)		Mean (SD)			n (%)		Mean (SD)			n (%)		Mean (SD)			n (%)		Mean (SD)	
Deceased-COVID	2 (1.8)		58.5 (4.9)			6 (3.1)		58.7 (6.2)			1 (0.2)		67.0 (0.0)			2 (0.2)		60.0 (4.2)	
Deceased-Other cause	1 (0.9)		70.0 (0.0)			0 (0)		NA			9 (1.7)		63.8 (10.7)			10 (1.2)		64.6 (7.8)	
Alive through 12/31/2020	108 (97.3)		57.8 (7.4)			186 (96.9)		59.0 (7.6)			524 (98.1)		59.1 (7.5)			794 (98.5)		60.6 (7.7)	
Proportion total deaths due to COVID	66.7%					100%					10.0%					16.7%			

Legend:

^a^VA Covid Shared Data Resource (CSDR) confirmed antigen/PCR test performed during study period resulting in definite test result; classified COVID+ if ever COVID+; and COVID^-^ if all test results confirmed negative.

^b^NDI death certificate file linked to CSP2006/MVP029 cohort; matching death certificate between 3/1/2020-12/31/2020 = deceased; COVID-related if COVID underlying or contributing cause of death; no matching death certificate ‘alive’ as of end study period.

^c^GWI case status determined using CDC (Fukuda) [8] severe symptom-based criteria based on CSP2006 GW Survey responses in deployed GWV subcohort; GWI^+^ meets criteria, otherwise GWI^-^.

Abbreviations:

CDC, Centers for Disease Control and Prevention

COVID^+^, VA PCR/antigen laboratory test confirmed definite COVID^+^ test finding between 3/1/2020-12/31/2020

COVID^-^, VA PCR/antigen laboratory test findings all confirmed definite COVID^-^

CSP2006, VA Cooperative Studies Program Gulf War Era Veteran (GWEV) cohort 2006 (also Million Veteran Program (MVP) cohort 029)

CSDR, VA’s COVID Shared Data Resource

DoD, U.S. Department of Defense

GWI, Gulf War Illness with GWI^+^, Gulf War Illness positive for severe CDC GWI criteria

GWI^-,^ Gulf War Illness absent as not meet CDC GWI criteria

GWEV, Gulf War Era Veteran with DoD confirmed military service n Gulf War era (08/02/1990-07/31/1991)

GWV, DoD confirmed deployment to PG conflict theater to serve in 1990–1991 Gulf War

MVP, Million Veteran Program.

NA, Not applicable

NDI, U.S. National Death Index

VA, U.S. Department of Veterans Affairs

#### COVID mortality burden associated with GWI in Deployed COVID-tested subcohort (N = 1,643).

There were only N = 11 death certificate-recorded COVID deaths in our COVID-tested deployed GWV subcohort, n = 8 among 303 confirmed-COVID^+^ and n = 3 among 1,340 confirmed-COVID^-^ (S6. Fig 3).

Among the *N* = 1,643 deployed GWVs with a completed 2018 Gulf War survey, the figure branches first by GWI status (GWI^+^ *N* = 645, 39%; GWI^−^ *N* = 998, 61%), then by COVID test result (COVID^+^ or COVID^−^), and finally by vital outcome (Alive, Died COVID,^d^ or Died Other Cause). GWI status was assigned using CDC Severe (Fukuda) criteria^e^ applied to 2018 survey responses. Footnote definitions are as in Fig 1 except: ^e^ Deployed GWV in subcohort classified using CDC Severe GWI criteria as GWI^+^ or GWI^−^ based on 2018 Gulf War survey responses.

GWI^+^ was not significantly associated with any metric of death certificate-based COVID-related mortality burden; with same very low proportion (0.2%) of GWI^+^ and GWI^-^ deployed GWVs who tested only COVID^-^ ultimately dying of COVID in 2020. (Table 4) In contrast, a non-significantly, slightly higher proportion of GWI^-^ deployed GWVs lab-confirmed COVID^+^ died from COVID (3.1% vs. 1.8% GWI^+^), with COVID accounting for non-significantly greater proportion of total deaths observed (100% [n = 6] in GWI^-^ vs. 67% [n = 2 of 3] in GWI^+^, respectively). (Table 4) Similar overall age-adjusted mortality rates (13.1/1,000 GWI^-^ vs. 11.7/1,000 GWI^+^, N.S.) were also observed, though CIs were imprecise given power constraints. (S3 Table)

## Discussion

Our study evaluated COVID-19 related health outcomes in a large nationwide cohort of 1990–1991 U.S. Gulf War veterans (GWVs), an aging group broadly exposed during military deployment to unique toxic exposures and with a high prevalence of Gulf War Illness (GWI^+^), a medically unexplained chronic multi-symptom illness associated with neuro-immune dysfunction and inflammation. We found robust, broadly reassuring results among COVID-tested veterans serving in the Gulf War era (GWEVs) using the VA healthcare system that overall deployment to the Persian Gulf (PG) theater was not associated with increased risks for testing COVID^+^ or experiencing COVID-related hospitalization, ICU admission, or death in the early 2020 pre-vaccine pandemic period.

We found that while GWI^+^ was also not associated with increased COVID test positivity (COVID^+^) among our deployed GWV subcohort, that GWI^+^ was associated with suggestive potential >2-fold significant increase in COVID-related hospitalization among deployed GWVs in both univariable and final multivariable models. These suggestive robust preliminary findings in our deployed GWV subcohort are, however, duly qualified pending replication in other large cohorts given fewer total hospitalizations limited power and confounder adjustment to designated core established COVID risk factors (i.e., age, race/ethnicity, sex, and Elixhauser comorbidity) only. In contrast, we found GWI^+^ was not associated with either strong or significant excess COVID-related ICU admission or mortality risk in deployed GWVs in preliminary unadjusted analyses, though even lower event rates precluded multivariable modeling.

The preliminary suggestive observed association between GWI^+^ and potential significantly increased COVID-related hospitalization in our deployed GWVs is perhaps reflective of a GWI-related vulnerability to more serious sequelae with COVID infection. This is biologically plausible given multiple epidemiological studies reported associations between GWI^+^ as well as overall GW deployment and increased prevalence of diverse chronic health conditions [3,4,8,37–39], with numerous experimental model, pre-clinical and clinical studies incorporating physiologic measures have documented significant neurological/neuropsychological [ 40–43], immunoinflammatory [44–46], metabolic [47–49], microbiome [50–52], and genetic differences associated with GWI^+^ [ 22,53–55]. It is also consistent with findings of increased susceptibility for adverse health effects in association with other exposures [ 56] among deployed GWVs who have GWI and multiple significant findings of long-term substantial increased health symptom burden [13,21] and frailty [57] and persistently reduced health-related quality of life [ 58] among GWI^+^.

Our *post-hoc* descriptive hypothesis generating bivariable exploration of 15 of most prevalent individual clinical comorbidities from among CDC evidence-based review identified risk factors for severe COVID and in Elixhauser comorbidity scores suggested two, coronary atherosclerotic heart disease (CAHD) and bronchitis, may potentially be differentially elevated among deployed GWI^+^ GWVs who experienced COVID-related hospitalization. The limited total number of hospitalizations, unfortunately, precluded further exploratory evaluation of them as additional potential independent risk factors in multivariable analyses after accounting for our other *a priori-specified* core COVID risk factors. However, both may warrant further investigation in future larger hypothesis testing cohort studies given: 1) GWI^+^ was recently associated with increased risk of CAHD in another GWV cohort [ 37]; and 2) chronic bronchitis and multiple other respiratory conditions (e.g., post-deployment asthma) are VA-recognized as presumptively military service-related among deployed GWVs given wide-scale exposure to airborne hazards including oil well fires and burn pits and epidemiological findings of increased occurrence of several respiratory diseases in deployed GWVs [ 59], with hazardous environmental airborne exposures also increasingly associated with greater COVID-related health risks in general population studies [ 60].

Another unique study contribution was our assessment of concordance between the VA CSDR-defined 30-day COVID-related mortality algorithmically ascertained using nearer term extracts from extant EHR and linked administrative healthcare databases against gold standard U.S. government official standard NDI death certificate data available only several years later. Excellent observed concordance provides important novel validation of a major VA CSDR-determined COVID outcome used in numerous high-impact published as well as ongoing studies on COVID risks and outcomes including on COVID vaccinations and post-acute long-term sequelae with COVID [ 61–64]. Notably, our NDI-based findings also reaffirmed our primary VA CSDR-determined multivariable relative risk findings of no significant association between deployment and 30-day COVID-related mortality among those testing COVID^+^. This suggests potential unmeasured selection bias was unlikely to have substantively impacted on our VA CSDR-based findings for overall deployment and COVID-related health outcomes.

Our study, which is to the best of our knowledge the first and only study to examine whether a specific veteran cohort deployed to serve in any military conflict (e.g., Vietnam War, 1990–1991 Gulf War, post-9/11 Iraq and Afghanistan conflicts) had potentially increased susceptibility to adverse COVID-related health outcomes had multiple methodological strengths. These include leveraging and expanding upon the well-phenotyped, largest nationwide research cohort of GWEVs, MVP’s CSP2006 Genomics of Gulf War Illness study cohort, which also includes the largest deployed GWV cohort with GWI case-status rigorously defined using National Academy of Sciences (formerly Institute of Medicine, NAS/IOM) recommended CDC criteria and conservative application of the severe phenotype. Additionally, GW survey responses used to determine contemporary GWI^+^ status were obtained from mid-2018 thru 2019, nearly contemporary to the pandemic period and thus a good marker for baseline susceptibility conveyed by GWI^+^, while also obtained long enough before the first U.S. confirmed case in January 2020 to mitigate potential reverse causation bias. Other strengths include utilizing the VA’s well-curated CSDR to comparably identify lab-confirmed COVID-testing/outcomes; our comprehensive methodologically-rigorous tiered multivariable analytic approach evaluating associations between overall deployment and GWI^+^ among deployed GWVs across range of COVID outcomes; and use of gold-standard NDI death certificate data as secondary source allowing us to interrogate age-adjusted COVID mortality cohort-wide (including in substantial number non-COVID tested GWEVs not included in CSDR database).

Importantly, our study is responsive to publicly expressed concerns of deployed GWVs, advocates and clinicians about possible elevated COVID risks given their military service-related toxic exposures and increased risk of several post-deployment exposure-related health conditions [ 65]. GWVs have long reported feelings of betrayal by the government, medical system, and even their country regarding under-recognized and non-redressed post-deployment military exposure-related health risks, burden and unmet healthcare needs [ 66] (including lack of clinical recognition of GWI^+^ both in VA and civilian healthcare settings and effective GWI^+^ tailored healthcare). Our study also aligns with the research and education aims included in The Sergeant First Class (SFC) Heath Robinson Honoring our Promise to Address Comprehensive Toxics (PACT) Act of 2022. The PACT Act has resulted in perhaps the largest expansion of VA healthcare system eligibility and benefits to date to include millions more potentially military service toxic exposed veterans deployed to serve in 1990−1991 GW, Vietnam, and post-9/11 Iraq and Afghanistan conflicts [ 67,68]. Post-deployment susceptibility to novel infectious agents, such as SARS-CoV-2, is not a presumed service-connected condition for any deployed veteran cohort; our findings do not support such a policy change for deployed GWVs.

Our study has several limitations, starting with the retrospective design that precludes causal inferences. Despite this being the largest nationwide research cohort of GWEVs, we had statistical power constraints for analyses of rarer COVID outcomes, particularly in our smaller deployed GWV subcohort analyses and associated power constraints in multivariable confounder adjusted risk modeling. This requires as previously noted qualified interpretation of preliminary findings of association between GWI^+^ and increased COVID-related hospitalization in deployed GWVs as suggestive only pending confirmation in other large external cohorts. Nonetheless, the general consistency in strength, direction and significance of relative risk estimates across unadjusted through all fully adjusted multivariable models, coupled with consistency in findings of no association between deployment or GWI^+^ and age-adjusted COVID-related mortality rates in both VA CSDR and NDI-based mortality analyses is supportive of internal validity of study findings. External generalizability is another important consideration; although earlier research evaluating the original CSP2006 parent cohort of >109,000 GWEVs demonstrated that it, as well as the large subgroup who returned 2018 Gulf War surveys, were generally representative in most sociodemographic and health characteristics to both the broader population of GWEVs using VHA healthcare and the full U.S. population of GWEVs [20]. Additionally, our observed COVID test positivity rates in our expanded CSP2006 cohort, which were similar across deployment and GWI case-status groups, were also aligned with reported COVID^+^ rates in COVID-tested adult males in the general US population in the early pandemic period [ 69]. Caution is still nonetheless warranted in extrapolating our findings to substantial number of GWVs not enrolled in VA healthcare system. VA-users are recognized as having higher rates of chronic illness and health risk factors and there are considerable differences between VA healthcare system (a highly subsidized, needs-based and prioritized equal access healthcare system) and civilian insurance-based healthcare systems with respect to access, utilization, and care delivery that could variably impact clinical outcomes. Our classification of contemporary, pre-pandemic Gulf War Illness (GWI^+^) status relied on a single time-point survey administered decades after the war ended, which may introduce limitations due to reliance on established IOM/NAS endorsed GWI^+^ research case definitions developed in much younger GWVs soon after the Gulf War ended. Despite this, prior analyses demonstrated that our surveyed deployed Gulf War veterans represent the broader national cohort of deployed GWVs in terms of key military service and sociodemographic characteristics [20,21]. Additionally, major risk factors identified for GWI^+^ in them were consistent whether using CDC severe phenotype (used in current COVID research), self-reported physician diagnosis of GWI^+^, or validated measures of multisymptom morbidity, and mirrored findings from previous and also much earlier large-scale studies [21]. This consistency suggests any potential misclassification bias is likely minimal and non-differential.

A further limitation is lack of direct chart review access within the VA COVID Shared Data Resource (CSDR) which prevented further valid characterization of symptomatic disease severity for algorithmically diagnostic code identified clinical comorbidities like bronchitis at time of COVID testing. Additionally, the absence of GWI-specific diagnostic codes precluded identification of GWI^+^ cases outside our deployed sub-cohort survey respondents. The recent introduction of first official diagnostic code for GWI [70], ICD-10 CM code T75.830 for GWI, along with Z77.31 for deployment-related Gulf War exposure (effective October 2025) [71] is expected to significantly enhance future EHR-based research. These codes should enable for the first time reliable, efficient identification of large representative samples of GWVs with comparable clinically documented GWI diagnosis seen across diverse healthcare settings, including importantly also in non-VA community care settings. This should greatly facilitate valid and reliable epidemiological assessment of even rarer outcomes—like COVID-19 related ICU admission and case fatality---within this high-risk population.

In conclusion, our study found overall deployment to the 1990–1991 Gulf War conflict theater was not associated with elevated risk of testing COVID^+^ or experiencing worse COVID-related health outcomes among Gulf War era veterans using the VA healthcare system in the early pre-vaccine pandemic period. Our preliminary suggestive findings of potential association between Gulf War Illness and increased COVID-related hospitalization among deployed Gulf War veterans warrant replication in other large external cohorts. Ongoing follow-up also needed to ascertain implications for our observed early pandemic-era associations with changes in viral variants, introduction of vaccines and effective oral anti-viral medication, and changes in persistence of symptoms associated with COVID infection.

## Supporting information

S1 Appendix VA Million Veteran Program Core Acknowledgements for Publications (October 2025). This appendix provides the required VA Million Veteran Program (MVP) core acknowledgements for publications using MVP resources, including names and institutional affiliations of the MVP Program Office, MVP Steering Committee, MVP Co-Principal Investigators, and MVP Core Operations leadership as of October 2025, as supplied by the MVP program office.
(PDF)

S2 Table Characteristics of N = 136,868 Gulf War era veterans (GWEVs) in the CSP2006 Million Veteran Program cohort by COVID test status in 2020. Sociodemographic and clinical characteristics of the full CSP2006/MVP029 GWEV cohort (N = 136,868) stratified by COVID lab-test status (never COVID-tested n = 110,727; ever COVID-tested n = 26,141) with further stratification by deployment status and confirmed COVID result (COVID⁺ n = 4,586; COVID⁻ n = 21,555), and the deployed GWV surveyed subcohort (N = 1,643) by COVID test status and Gulf War Illness (GWI) case status. Variables presented include age, sex, race/ethnicity, marital status, military rank, service branch, unit component, smoking history, Elixhauser comorbidity score, BMI, co-insurance, VA healthcare utilization, and geographic region. Cell values are n (%).
(XLSX)

S3 Table COVID-related mortality rates in the CSP2006/MVP029 Gulf War era veteran cohort by deployment status, Gulf War Illness status, and COVID lab-test result. Age-adjusted COVID-related mortality rates per 1,000 persons and mortality rate ratios (deployed vs. non-deployed) in three sections: (A) lab-confirmed COVID⁺ in the overall CSP2006 cohort (N = 4,586); (B) lab-confirmed COVID⁻ (N = 21,555); and (C) the deployed GWV surveyed subcohort (N = 1,643) by COVID test status and GWI case status (GWI ⁺ /GWI⁻). Age strata are 47–59, 60–69, and ≥70 years. Rates and rate ratios are presented with 95% confidence intervals.
(XLSX)

S4 Text Supplemental Results: Secondary National Death Index analyses and pre-pandemic versus early-pandemic mortality comparison. Two secondary analyses: (1) concordance between VA COVID Shared Data Resource (CSDR) and National Death Index (NDI) COVID-related mortality ascertainment—among 103 VA CSDR-identified COVID decedents, 100 (97%) had NDI-matched death certificates recording COVID as a cause of death (92% as the underlying cause), with no additional COVID deaths identified solely through NDI; (2) comparison of cohort all-cause mortality between pre-pandemic year 2019 (N = 138,058; 1,191 deaths; 99.1% survival) and the early pandemic period March–December 2020 (N = 136,868; 1,556 deaths; 98.9% survival).
(DOCX)

S5 Fig Vital outcomes among CSP2006 Gulf War era veterans never lab-tested for COVID in 2020, by deployment status. Flow diagram showing vital outcomes through 12/31/2020 for the N = 110,727 GWEVs not lab-tested for COVID during the study period (cross-referenced from Fig 1). Veterans are stratified by deployment status (Deployed N = 24,178, 22%; Non-Deployed N = 86,549, 78%) and vital outcome (Alive, Died COVID,ᵈ or Died Other Cause). The N = 26,141 ever COVID-tested are cross-referenced to Fig 1. Footnote definitions as in Fig 1.
(TIF)

S6 Fig COVID-19 lab-test status and vital outcomes among the deployed Gulf War veteran surveyed subcohort, stratified by Gulf War Illness status. Flow diagram showing COVID lab-test result and vital outcomes through 12/31/2020 for the deployed GWV surveyed subcohort (N = 1,643), stratified by GWI case status: GWI⁺ (N = 645, 39%) and GWI⁻ (N = 998, 61%). Within each GWI stratum, veterans are further stratified by COVID test result (COVID⁺ or COVID⁻) and vital outcome (Alive, Died COVID,ᵈ or Died Other Cause). GWI case status classified using CDC Severe (Fukuda) criteriaᵉ applied to 2018 Gulf War survey responses. Footnote definitions as in Fig 1.
(TIF)

## Abbreviations

ADJ: adjusted
CAHD: coronary atherosclerotic heart disease
CDC: Centers for Disease Control and Prevention
CI: Confidence interval
CIPHER: Centralized Interactive Phenomics Resource
COVID^+^: any VA PCR laboratory test confirmed definite COVID+ test finding between 3/1/2020-12/31/2020
COVID^-^: VA laboratory test findings all confirmed definite COVID negative
CSP2006: VA Cooperative Studies Program Genomics of Gulf War Illness study cohort (alsoMillion Veteran Program (MVP) 029)
CSDR: VA’s COVID Shared Data Resource
DoD: U.S. Department of Defense
GWI^+^: Gulf War Illness positive based on meeting CDC severe GWI criteria
GWI^-^: Gulf War Illness absent as not meeting CDC GWI criteria
GWEV: veteran with confirmed military service in the 1990-1991 Gulf War era period
GWV: GWEV deployed to Persian Gulf conflict theater to serve in 1990-1991 Gulf War
IOM: Institute of Medicine
ICU: intensive care unit
MRR: mortality rate ratio
NAS: National Academy of Sciences
NDI: National Death Index
N.S.: not statistically significant
OR: Odds Ratio
PCR: polymerase chain reaction
PG: Persian Gulf
MVP: VA Million Veteran Program
VA: U.S. Department of Veterans Affairs
