# Supplementary material for: Association between deployment and Gulf War Illness and adverse COVID outcomes in a nationwide cohort of 1990–1991 Gulf Era war veterans in the VA’s Million Veteran Program
Source: PLoS One. 2026 Jun 15;21(6):e0348594. doi: 10.1371/journal.pone.0348594 (PMC13268159; doi:10.1371/journal.pone.0348594)
Supplement: S4 Text — Two secondary analyses: (1) concordance between VA COVID Shared Data Resource (CSDR) and National Death Index (NDI) COVID-related mortality ascertainment—among 103 VA CSDR-identified COVID decedents, 100 (97%) had NDI-matched death certificates recording COVID as a cause of death (92% as the underlying cause), with no additional COVID deaths identified solely through NDI; (2) comparison of cohort all-cause mortality between pre-pandemic year 2019 (N = 138,058; 1,191 deaths; 99.1% survival) and the early pandemic period March–December 2020 (N = 136,868; 1,556 deaths; 98.9% survival). (DOCX) [file pone.0348594.s004.docx]

**S4 Text. Supplemental Results**

*Secondary NDI Analyses:*

NDI and VA CSDR COVID-related mortality concordance: All 103 VA CSDR-identified COVID decedents (i.e., EHR database identified death occurred ≤30-days of lab-confirmed COVID^+^) had NDI official matching death certificates, 100 (97%) with COVID recorded as a cause of death (92% of whom it was listed as the underlying cause). No additional COVID-related were identified solely by NDI searches (i.e., no additional deaths where death certificate recorded COVID occurred >30-days after lab-confirmed COVID^+^).

Mortality comparison 2019 (pre-pandemic) and early pandemic period (3/1/2020-12/31/2020): Our NDI searches identified 1,191 total deaths in our CSP2006 GWEV cohort confirmed alive through 12/31/2018 (N=138,058) occurred in between 1/1/2019-12/31/2019 (pre-U.S. COVID pandemic), with 99.1% overall 12-month cohort survival in 2019. In comparison, 1,556 total deaths occurred in our COVID study eligible CSP2006 cohort (N=136,868) between 3/2020-12/2020 (see Fig 1 and S5 Fig), with 98.9% overall 10-month cohort survival.
