# Supplementary figures and images for: Association between deployment and Gulf War Illness and adverse COVID outcomes in a nationwide cohort of 1990–1991 Gulf Era war veterans in the VA’s Million Veteran Program
Source: PLoS One. 2026 Jun 15;21(6):e0348594. doi: 10.1371/journal.pone.0348594 (PMC13268159; doi:10.1371/journal.pone.0348594)

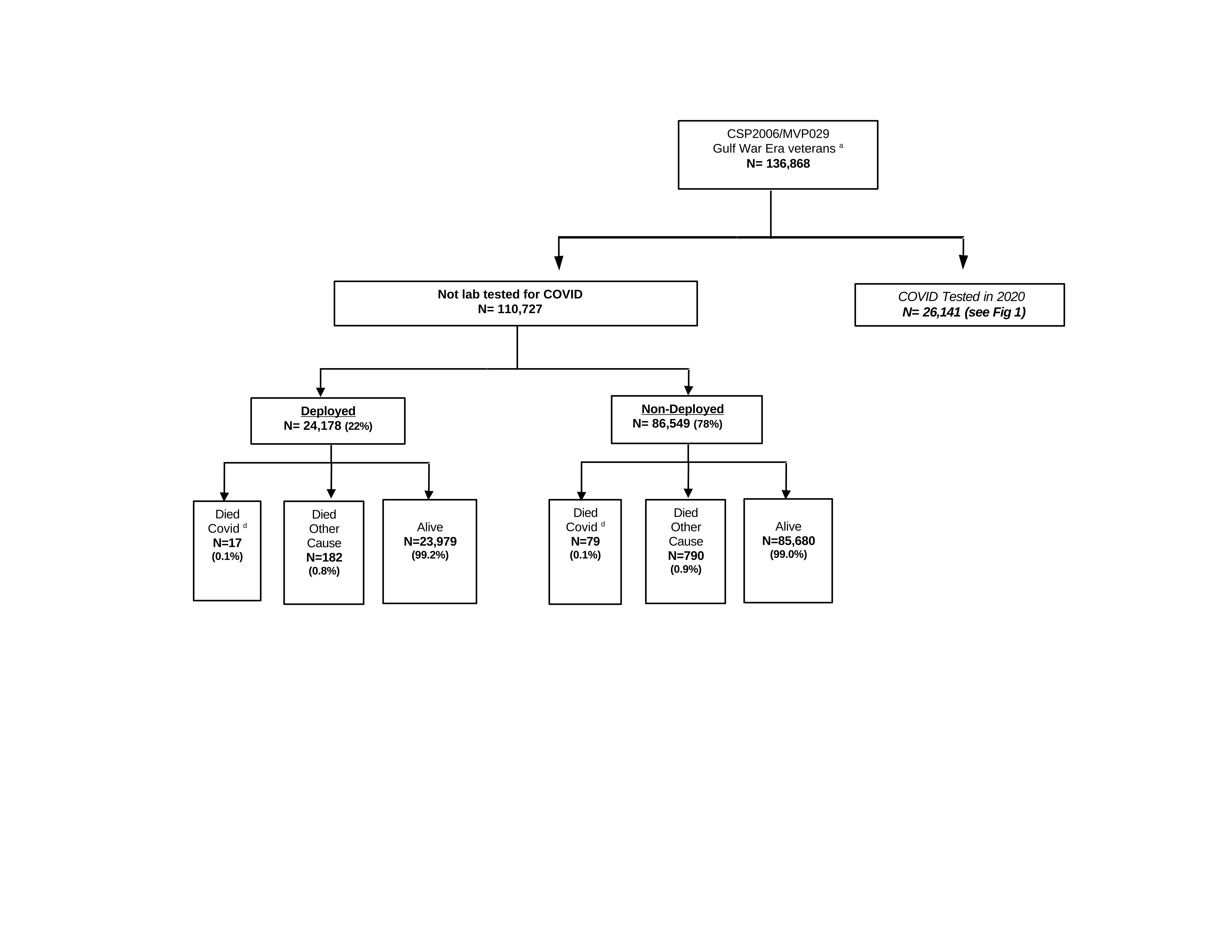

Supplement: S5 Fig — Flow diagram showing vital outcomes through 12/31/2020 for the N = 110,727 GWEVs not lab-tested for COVID during the study period (cross-referenced from Fig 1). Veterans are stratified by deployment status (Deployed N = 24,178, 22%; Non-Deployed N = 86,549, 78%) and vital outcome (Alive, Died COVID,ᵈ or Died Other Cause). The N = 26,141 ever COVID-tested are cross-referenced to Fig 1. Footnote definitions as in Fig 1. (TIF) [file pone.0348594.s005.tif]

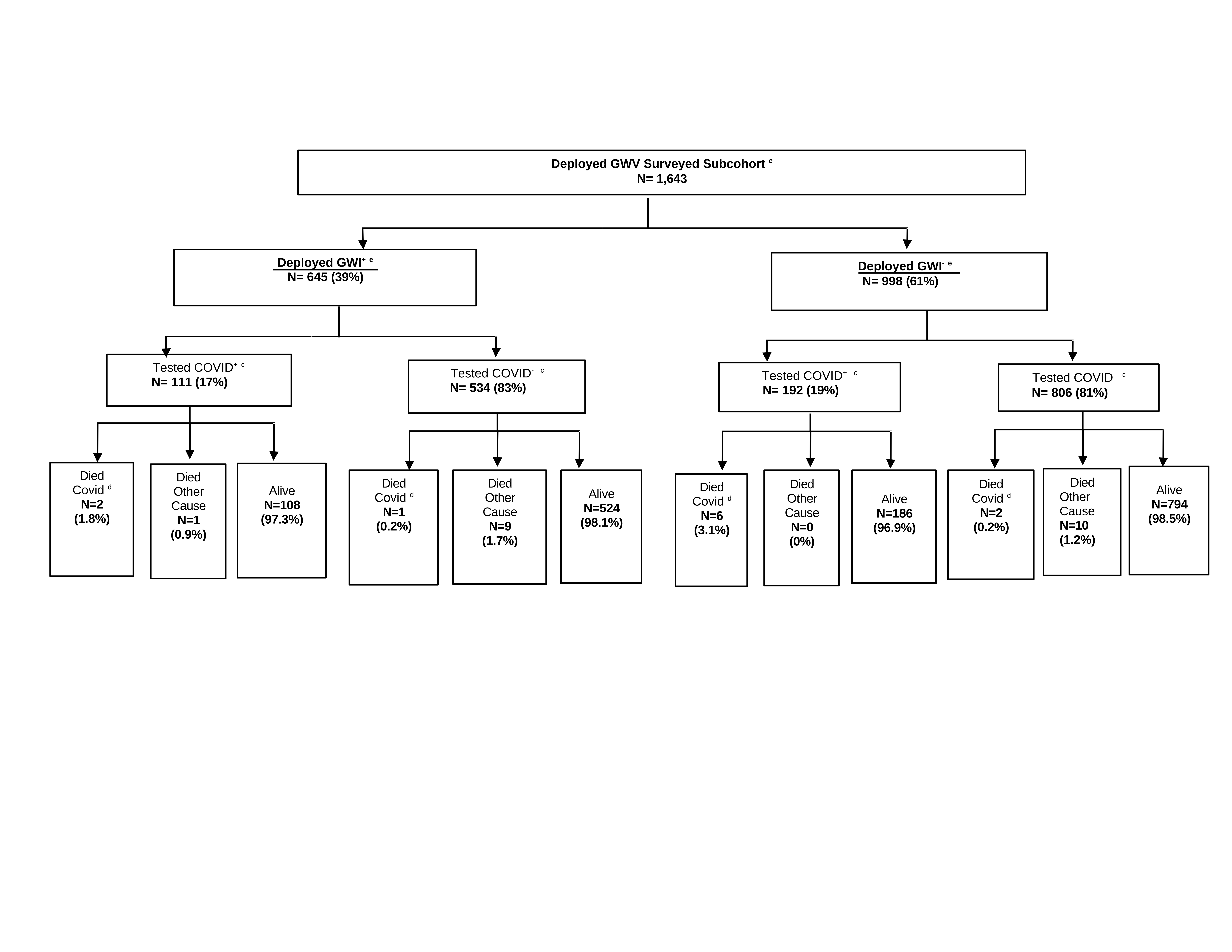

Supplement: S6 Fig — Flow diagram showing COVID lab-test result and vital outcomes through 12/31/2020 for the deployed GWV surveyed subcohort (N = 1,643), stratified by GWI case status: GWI⁺ (N = 645, 39%) and GWI⁻ (N = 998, 61%). Within each GWI stratum, veterans are further stratified by COVID test result (COVID⁺ or COVID⁻) and vital outcome (Alive, Died COVID,ᵈ or Died Other Cause). GWI case status classified using CDC Severe (Fukuda) criteriaᵉ applied to 2018 Gulf War survey responses. Footnote definitions as in Fig 1. (TIF) [file pone.0348594.s006.tif]
